# Editorial: Molecular aspects of compulsive drug use

**DOI:** 10.3389/fpsyt.2023.1252507

**Published:** 2023-07-25

**Authors:** Amy W. Lasek, Daniel da Silva, Doo-Sup Choi

**Affiliations:** ^1^Department of Pharmacology and Toxicology, Virginia Commonwealth University, Richmond, VA, United States; ^2^Icahn School of Medicine at Mount Sinai, New York, NY, United States; ^3^Department of Molecular Pharmacology and Experimental Therapeutics, Mayo Clinic College of Medicine and Science, Rochester, MN, United States

**Keywords:** alcohol use disorder, methamphetamine, cocaine, epigenetic, sex differences, miRNA - microRNA, gene by environment (GxE) interaction, aversion-resistant drinking

Drug addiction is a complex psychiatric disorder defined by a compulsion to seek and take the drug, losing control over intake, and continuing to take the drug despite negative consequences. The inability to limit drug consumption leads to relapse and failure in treatment, thus understanding the underlying molecular mechanisms that contribute to compulsive drug seeking and taking is critical to developing effective treatments. The medical term for drug addiction is substance use disorder (SUD), which is defined by an individual having two or more of the 11 criteria that are outlined in the 5th edition of the American Psychiatric Association (1) Diagnostic and Statistical Manual of Mental Disorders (DSM-5). Several criteria for SUD encompass a wide range of indicators that associate with compulsive drug use. These criteria specifically focus on two key aspects: risky use and social impairment. Risky use is characterized by recurrent substance use in physically unsafe environments and persistent substance use despite awareness of potential physical or psychological harm. Social impairment criteria encompass the inability to fulfill major obligations at work, school, or home; continued use of the substance despite significant social or interpersonal problems; and reduction or discontinuation of recreational, social, or occupational activities due to substance use. Despite significant research efforts, the precise molecular mechanisms underlying compulsive drug use in remain largely unknown. However, gaining a deeper understanding of the neurobiological mechanisms that underly compulsive drug taking could lead to novel pharmacological and psychological tools to treat SUD. The papers in this Research Topic, “*Molecular aspects of compulsive drug use*” provide new knowledge toward achieving this goal. This collection contains preclinical and clinical studies on three different substances: alcohol, cocaine, and methamphetamine. Three of the papers on this topic also have a secondary emphasis on sex differences in compulsive drug use. Women have historically been underrepresented in behavioral neuroscience research but like men, also suffer from SUD. Understanding differences in neurobiology that contribute to sex differences in compulsive drug use will aid in developing effective treatments for both sexes.

## Compulsive alcohol drinking

As in any type of behavioral neuroscience research, the development of animal models is an essential first step to understanding the underlying molecular neurobiology. The review article by De Oliveira Sergio et al. compares two commonly used animal models of compulsive-like alcohol drinking (CLAD): foot shock-resistant operant ethanol self-administration and quinine-resistant alcohol drinking in the home cage. In the foot shock procedure, animals must overcome a painful foot shock to obtain an alcohol reward. In the quinine-resistant alcohol drinking procedure, bitter-tasting quinine is added to the alcohol solution; animals exhibit CLAD when they continue to consume alcohol even though it contains quinine. Previous studies by the Hopf lab [De Oliveira Sergio et al.; (2, 3)] have demonstrated that prefrontal cortex and anterior insular cortex (AIC) circuits are involved in CLAD using both the foot shock- and quinine-resistant models of alcohol drinking. In the review article (De Oliveira Sergio et al.), they argue for the utility of the quinine-resistant alcohol consumption model, given its ease of use, ability to test multiple doses of quinine and perform repeated testing within subjects. The article also describes the current state of the literature on sex differences in aversion-resistant alcohol consumption. They conclude that many, but not all, studies have shown that female rats and mice are more aversion-resistant than males in the quinine- and foot shock-resistant models of CLAD. Finally, they touch on neural mechanisms involved in CLAD and pharmacological strategies to reduce this behavior, including the use of α1 and β adrenergic receptor antagonists (3) that could be repurposed for use in individuals with alcohol use disorder (AUD).

In terms of sex differences in CLAD and the role of the AIC in this behavior, the paper by Martins de Carvalho et al. studied the involvement of specialized extracellular matrix structures called perineuronal nets (PNNs) in the AIC of male and female mice in CLAD using the quinine-resistant alcohol drinking model. They found that female mice were more resistant to the addition of quinine in alcohol than male mice, despite being equally sensitive to the aversive taste of quinine in water. Female mice had stronger staining of PNNs in the AIC than males, suggesting that this might be contributing to sex differences in CLAD. However, disrupting PNNs in the AIC by microinjecting an enzyme that breaks down the glycosaminoglycan components of PNNs had almost no effect on CLAD in female mice, whereas disrupting PNNs in the AIC of male mice decreased CLAD. These results indicate that molecular and cellular mechanisms driving CLAD differ between the sexes.

The paper by Sneddon et al. begins to tease apart the biological mechanisms involved in sex differences in CLAD. The authors investigated whether sex chromosome complement (XX or XY) contributes to binge-like alcohol consumption in a two-bottle choice drinking in the dark (DID) test and CLAD using the quinine-resistant model of alcohol consumption. They employed the four-core genotypes (FCG) mouse model, in which the testes-determining gene *Sry* has been moved from the Y chromosome to an autosome (4). Mating XY/Sry+ males with XX females generates four genotypes: XY/*Sry*+ (testes), XX/*Sry*+ (testes), XY/*Sry*- (ovaries), and XX/*Sry*- (ovaries). This allows testing of sex chromosome effects on the brain and behavior independent of gonadal phenotype. The main conclusions (Sneddon et al.) were that XY chromosomal complement in *Sry*+ mice resulted in increased consumption and preference for alcohol in the DID test, and that XY chromosomal complement in *Sry*- mice resulted in aversion-resistance in the CLAD test. Another important finding was that *Sry*+ mice were insensitive to quinine in water. Although gonadal hormones have been implicated in alcohol drinking in several studies (5), this paper demonstrates that sex chromosomes can also contribute to binge-like drinking and CLAD.

## Epigenetic mechanisms in cocaine sensitization and methamphetamine use disorder

The paper by Parsegian et al. examined gene x environment interactions in sensitivity to the psychomotor activating effects of cocaine and associated epigenetic modifications on histone H3 protein in rat brain. The authors used a genetic model of differential locomotor response to a novel environment, the bred high responder (bHR) and bred low responder (bLR) lines of rats, which also exhibit differences in addiction-related behaviors (6). Rats were given cocaine injections during adolescence when the brain is still developing and a vulnerable period in which drug exposure can increase the risk of developing substance use disorder as an adult (7). The authors (Parsegian et al.) used a cocaine sensitization procedure, a model of behavioral plasticity in which animals develop an enhanced locomotor response to cocaine after repeated injections compared to the response to a single injection. Rats were tested for cocaine responses in adulthood after adolescent cocaine exposure. They found that genetic background modified the effect of adolescent cocaine exposure on the psychomotor response to cocaine in adulthood and histone H3 lysine 9 trimethylation (a repressive epigenetic mark) and acetylation (a permissive epigenetic mark) in the nucleus accumbens. This paper underscores the importance of genetic background on influencing epigenetic and behavioral responses to cocaine exposure during a critical developmental period.

The translational paper by Xu et al. examined another type of epigenetic factor, microRNAs (miRNAs), as biomarkers for methamphetamine use disorder (MUD) in humans. The study focused on a specific family of miRNAs named miR-320 in the plasma and exosomes of individuals with MUD compared to control subjects. Individuals with MUD had increased miR-320 in the plasma and exosomes. The authors also found that levels of miR-320 in exosomes could predict MUD. As miRNAs regulate gene expression by enhancing degradation or inhibiting translation of target mRNAs (8), the authors also performed bioinformatic analysis to identify potential miR-320 target mRNAs. This study provides preliminary evidence that miR-320 could be used as an objective measure of MUD and identifies potential new targets of miR-320 that should be followed up in animal models of methamphetamine addiction.

This Research Topic provides an important basis for future work on the compulsive aspects of SUD and will hopefully inspire and encourage further investigation into identifying new molecular targets to treat SUD.

## Author contributions

AL wrote the first draft. DS and D-SC edited the article. All authors contributed to the article and approved the submitted version.
